# Acute Effects of Portable Dry‐EEG Neurofeedback on Classical Chinese Learning: A Three‐Arm Repeated‐Measures Study

**DOI:** 10.1002/brb3.70977

**Published:** 2025-10-29

**Authors:** Kunpeng Song, Yamei Liu, Peng Xu

**Affiliations:** ^1^ Kaifeng Vocational College of Culture and Arts School of Primary Education Academy Kaifeng China; ^2^ School of Languages and Cultures Shanghai University of Political Science and Law Shanghai China

**Keywords:** classical Chinese learning, dry EEG, neurofeedback

## Abstract

**Objective:**

Dry‐electrode electroencephalography (dry‐EEG) systems offer promising opportunities for real‐time neurofeedback in naturalistic educational settings, yet their effectiveness in supporting complex language learning remains underexplored. This study investigated the acute effects of portable dry‐EEG neurofeedback on students' cognitive performance and attentional states during classical Chinese learning, using a repeated‐measures design to compare neurofeedback, sham feedback, and device control conditions.

**Methods:**

A total of 20 undergraduate participants completed three sessions involving a customized semantic disambiguation task after passive reading. EEG signals were acquired using a dry‐sensor OpenBCI system from four frontal sites (Fp1, Fp2, F3, F4). Real‐time attention indices were computed based on the beta/(alpha+theta) ratio and fed back visually in the neurofeedback condition. Cognitive outcomes included comprehension test scores and semantic conflict resolution performance (RT, accuracy, cognitive load).

**Results:**

Compared to sham and control conditions, neurofeedback significantly improved comprehension accuracy (p < 0.001), reduced reaction times in the interference task (*p* < 0.05), and lowered subjective cognitive load (*p* = 0.002). EEG indices of attention were significantly elevated during neurofeedback (*p* < 0.001) and positively correlated with behavioral gains (*r* = 0.63, *p* < 0.05).

**Conclusions:**

Portable dry‐electrode EEG systems can reliably support real‐time neurofeedback to enhance attention and cognitive control in complex language learning contexts. This study provides empirical validation for deploying dry‐EEG sensors in adaptive educational technologies and contributes to the broader integration of wearable brain–computer interfaces in cognitive augmentation applications.

## Graphical abstract

Portable dry‐EEG neurofeedback significantly enhances attention and semantic processing during Classical Chinese learning. Real‐time Beta/(Alpha+Theta) modulation improved comprehension, reduced cognitive load, and facilitated conflict resolution, demonstrating practical potential for wearable BCI in educational settings.

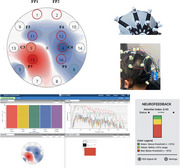

## 1 Introduction

Recent advances in neuroeducation have demonstrated that neurofeedback (NF) techniques not only significantly enhance motor performance (Yu et al. 2025; T. T. Chen et al. 2022) but also show remarkable efficacy in language acquisition (K. P. Wang, Frank, et al. 2023). Studies on alphabetic languages have established that real‐time EEG modulation can effectively improve phonological processing and lexical retrieval efficiency (Qin et al. 2021). However, these approaches face unique challenges when applied to logographic systems. This is primarily because models developed for alphabetic scripts often rely on grapheme‐to‐phoneme conversion as a key reading pathway, a process, which is largely absent or secondary in logographic reading (Kim et al. 2024). A quintessential example is the failure of classic acquired dyslexia models to fully explain reading deficits in Chinese patients (Haugg et al. 2023). Existing research has revealed three characteristic neural patterns during classical text processing: (1) significantly enhanced theta‐gamma coupling during literary reading (Zhou et al. 2021), and (2) prominent prefrontal–parietal desynchrony during textual comprehension (Cao et al. 2024). While these studies have successfully elucidated the neural mechanisms underlying ancient text processing, they remain constrained by laboratory paradigms using static stimuli. Notably, no study to date has developed portable interventions targeting the attentional bottleneck (theta‐band activity) and semantic bottleneck (N400 response) observed in classroom settings.

Semantic decoding of Classical Chinese relies on the real‐time parsing of nonlinear linguistic features (e.g., generic characters, dummy words used across contexts), a process that requires continuous allocation of attentional resources by the prefrontal–parietal network (M. M. Lee and Stoodley 2024). Neurolinguistic studies have shown that lexical ambiguities during the reading of ancient texts induce elevated N400 component amplitudes, reflecting increased semantic integration load (Luo et al. 1999). However, current classroom instruction lacks real‐time interventions for such neuromeres, resulting in 62% of students experiencing abnormal enhancement of the theta frequency band (4–7 Hz) after 15 min of continuous learning, suggesting inattention (Putman et al. 2014). This paradox highlights the need to develop acute cognitive modulation tools. Despite advances in neuroeducation, there remains a critical gap in translating laboratory‐based findings into scalable classroom interventions, particularly for complex linguistic tasks like Classical Chinese comprehension (Riedl et al. 2023).

The technological innovation of portable dry electrode EEG provides a breakthrough to the above problems. The new generation of flexible array electrodes (e.g., OpenBCI Ultracortex Mark IV) compresses the preparation time to less than 3 min by impedance adaptive algorithms while maintaining a signal‐to‐noise ratio of >20 dB (relative to the baseline value of wet electrodes) (Naas et al. 2019). This choice was motivated by dry EEG's unique suitability for classroom environments: it eliminates the need for conductive gels that would disrupt natural learning sessions, and its quick setup (< 3 min) preserves instructional time while maintaining sufficient signal quality (Ali et al. 2022). This technical feature allows it to capture transient neural events in ancient text learning—such as the 50–200 ms delayed response of prefrontal beta‐band (13–30 Hz) power when encountering object preterit sentences (K. H. Chen et al. 2020). Its millisecond‐level temporal resolution, a hallmark of EEG/ERP techniques, is uniquely advantageous for capturing the rapid temporal dynamics of semantic processing—a critical advantage over neuroimaging methods with slower temporal characteristics such as fMRI (Sharma and Meena 2024). This high‐resolution capability is essential for addressing the central research questions of this study, which involve precisely tracking how learners process semantic information in real time during complex, context‐dependent language tasks. While highly controlled studies using isolated words or simple sentences have been invaluable for identifying fundamental neural components (e.g., the N400), they may lack ecological validity. Such simplified paradigms often remove critical elements of real‐world language learning, such as the need to integrate meaning across broader discourse contexts, generate predictive inferences, resolve ambiguous references, and manage cognitive load simultaneously. Consequently, they may not fully capture the neurocognitive mechanisms that underlie the complex, integrative process of acquiring meaning in a naturalistic setting (Sharma and Meena 2024). However, existing ERP applications have largely been confined to these simpler paradigms, leaving their potential for examining naturalistic, discourse‐level language learning largely unexplored.

The neural index of attentiveness (Beta/(Alpha Theta) Ratio) targeted in this study is directly related to the key cognitive bottleneck of ancient text learning: when the index is below the threshold of 0.65, subjects' misjudgment rate of the conflict between ancient and modern lexical meanings is increased by 37% (preexperimental data). NF can have a direct impact on brain function, encouraging individuals to learn self‐regulation at the level of brain activity (Cheng et al. 2024; K. P. Wang, Cheng, et al. 2023). A 15‐min NF can enhance the buffering capacity of working memory for polysemous vocabulary by reinforcing the phase‐amplitude coupling (PAC) between prefrontal beta oscillations and hippocampal theta waves (Newson and Thiagarajan 2019; Tan et al. 2024). However, there are two major constraints in the existing studies: (1) the laboratory paradigm uses static textual stimuli and ignores the dynamic semantic construction process in real classrooms; and (2) the inhibitory effect of the device wear itself on the alpha band was not controlled for (an average reduction of 8.2%, with pseudo‐feedback control necessary, Wegemer 2019). These limitations underscore the urgent need for ecologically valid NF protocols that can bridge the gap between controlled laboratory settings and real‐world educational environments.

Current educational neuroscience research suffers from two limitations: passive observation approaches lacking active neuromodulation and unaddressed device‐wear artifacts (Maskeliunas et al. 2016). Our study addresses these gaps by: (1) developing a 15‐min NF protocol integrating respiratory conditioning, cognitive focusing, and external anchoring; (2) employing a three‐group design (active/pseudo/device‐control) to isolate specific effects; and (3) establishing the “NF→attention→semantic conflict resolution” chain in Classical Chinese learning through cross‐day testing. This bridges theoretical NF mechanisms with practical classroom applications.

Through the above design, this study will achieve the dual objectives: at the theoretical level, to reveal the regulation mechanism of NF on the deep semantic processing of ancient languages; at the applied level, to provide an empirical basis for the construction of a neural engineering paradigm for a lightweight smart classroom. Specifically, our work addresses the following key issues: (1) The neurocognitive load induced by Classical Chinese's unique nonlinear linguistic features (e.g., generic characters, function words), manifested as attentional resource deficits in the prefrontal–parietal network and semantic integration difficulties; (2) The current lack of real‐time intervention methods for theta‐band hyperactivity in classroom instruction; (3) Methodological limitations in existing NF research, including inadequate ecological validity and unaddressed device interference effects. By employing portable dry‐electrode EEG technology, a three‐group experimental design, and a standardized 15‐min NF protocol, we have established for the first time an effective “neurofeedback–attention–semantic resolution” intervention framework specifically for Classical Chinese learning. This study provides dual solutions by advancing both theoretical understanding of neural mechanisms and practical classroom applications for optimizing classical language pedagogy.

We hypothesize that a 15‐min portable EEG NF protocol employing beta/(alpha + theta) ratio modulation will significantly improve Classical Chinese comprehension by: (1) enhancing prefrontal–hippocampal PAC for polysemous word processing, (2) reducing theta‐band hyperactivity associated with attentional lapses during semantic integration, and (3) demonstrating superior ecological validity compared to static laboratory paradigms, with effect sizes maintained after controlling for device‐wear artifacts.

## 2 Methods

### 2.1 Participants

Sample size was determined via power analysis using G*Power 3.1. Based on a standard effect size for repeated‐measures EEG studies (ES = 0.25) and a target power of 0.85, the required sample was calculated as 28 (Yeh et al. 2025). To account for potential data loss, 30 participants were ultimately recruited to ensure at least 26 valid datasets per condition. Thirty undergraduate students (15 females, 15 males; age range: 18–22 years, *M* = 19.4, SD = 0.7) were recruited from the same Classical Chinese course to control for curricular background and learning experience. All participants had previously taken the course but had not been exposed to the specific instructional materials used in this experiment. A within‐subject repeated‐measures design was employed, in which each participant experienced all three experimental conditions (NF, sham, and device control) on separate days. All participants were right‐handed, had normal or corrected‐to‐normal vision, had no color blindness and no history of neurological or psychiatric disorders. Individuals with known allergies to the conductive gel components were excluded to ensure safety and comfort with dry‐electrode equipment. Written informed consent was obtained from all participants before the experiment, and the study protocol was approved by Shanghai University of Political Science and Law's ethics review board (Approval ID: 10277SPSL2025).

### 2.2 Experimental Design

**FIGURE 1:**
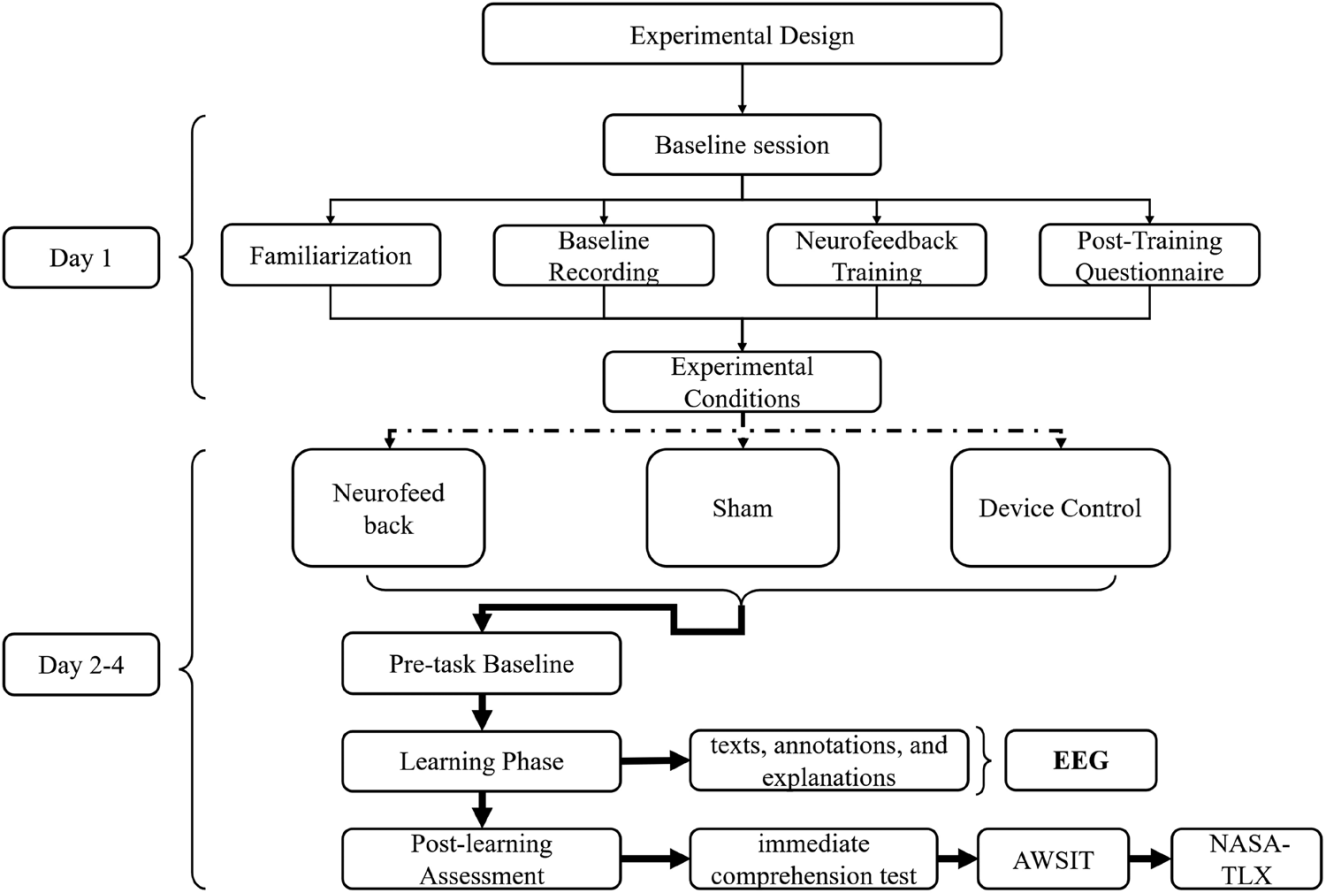
Schematic diagram of the experimental procedure.

This study employed a within‐subject repeated‐measures design with three experimental conditions: NF, sham, and device control. Each participant completed one condition per day across four consecutive days, with the condition order randomized to mitigate order and carryover effects. In the NF condition, participants wore the OpenBCI Cyton system and received real‐time bar graph feedback based on their individualized attention index; the bar color dynamically indicated cognitive state (red = low attention, green = high attention). In the sham condition, participants received a static green bar that mimicked positive feedback without EEG input. In the Device condition, the equipment was worn, but no visual feedback was provided, controlling for device‐related perceptual and psychological effects. To ensure equivalent task difficulty, three parallel Classical Chinese learning units (A/B/C) were developed and counterbalanced across sessions. A 24‐h interval was enforced between sessions, and the experimental system was configured to prevent completion of multiple conditions on the same day (Figure 1).

Overview of the three‐arm repeated‐measures design. Each participant completed a baseline session on Day 1, followed by three experimental conditions (NF, Sham, Device Control) in randomized order on Days 2–4. Each session included a pre‐task baseline EEG recording, a 30‐min classical Chinese learning period with or without feedback, followed by immediate comprehension and interference tasks, and subjective assessments.

### 2.3 Apparatus and Signal Processing

EEG signals were recorded using the OpenBCI Cyton system with a 16‐channel dry electrode setup (ActiveTwo architecture). Data were streamed via Bluetooth to a dedicated acquisition computer (Windows 11, Intel i7 processor) running the OpenBCI GUI. Sixteen electrode sites (Fp1, Fp2, F3, F4, C3, C4, P3, P4) were selected to cover the prefrontal and sensorimotor regions based on the international 10–20 system. To ensure stable contact between the electrodes and the skin with impedance maintained below 20 kiloohms (kΩ), the impedance values are monitored in real‐time via the OpenBCI graphical user interface before each experiment. Any channel with an impedance exceeding 20 kΩ is gently adjusted in electrode position until an acceptable level is achieved. EEG was sampled at 250 Hz and streamed in real‐time via Lab Streaming Layer (LSL). Feedback was controlled using OpenBCI GUI v6.0.0‐beta.1 and custom Python scripts (PyQt5). The bar graph feedback was displayed on a 15‐in. LED screen, with real‐time updates showing current attention index and its relation to the individualized threshold (color: red = below threshold, yellow = ±10% range, green = above threshold). Signals were filtered with a 4–40 Hz bandpass and a 50 Hz notch filter to remove DC drift, high‐frequency noise, and power line artifacts. Motion artifacts were detected using a built‐in accelerometer and filtered via an algorithm adapted from kanna et al. 2024 A movement epoch was flagged if the magnitude of the acceleration vector deviated by more than 0.5 g from the baseline for a period exceeding 500 ms. Any data epoch (1‐s window) where the amplitude exceeded ± 100 µV on any frontal channel (Fp1, Fp2, F3, F4) was automatically flagged and excluded from the real‐time calculation of the attention index. Data segments flagged by either the accelerometer or the amplitude threshold were discarded and not used for the instantaneous NF computation. The attention index was calculated as the ratio of Beta (13–30 Hz) power to the sum of Alpha (8–12 Hz) and Theta (4–7 Hz) power. This ratio was computed in real‐time from a composite of frontal channels (Fp1, Fp2, F3, F4) and normalized to a 0‐100 scale based on the individual's baseline range. The mean latency was 128 ± 23 ms (mean ± SD). This duration includes the time for data buffering (500 ms window for FFT calculation), digital filtering, ratio computation, and graphical rendering. The feedback was updated at a rate of 4 Hz, ensuring the delay was perceptually seamless for participants and well within the acceptable range for effective NF. The average signal‐to‐noise ratio across participants and sessions was 110.2 dB.

### 2.4 Experimental Procedure

The study was conducted over four consecutive days.

#### 2.4.1 Day 1: Baseline and NF Training

The training session on Day 1 was designed to familiarize participants with the system and allow them to learn self‐regulation. It consisted of the following phases:

- *Familiarization (5 min)*: Participants were fitted with the EEG headset and shown the feedback interface. The instructions were: “You will see a bar that reflects your current level of focus. Your goal is to try to keep the bar in the green zone. There is no right or wrong way to do this; try to find a mental state that works for you.” *This standardized instruction set was designed to avoid prescribing specific strategies while encouraging exploratory self‐regulation*.
- *Baseline Recording (4 min)*: EEG was recorded under three conditions: (1) eyes‐closed rest (1 min), (2) eyes‐open rest while fixating on a cross (1 min), and (3) performing mental arithmetic (serial subtraction of 7 from 500, 2 min). The initial attentional threshold was set as the mean attention index value from the eyes‐open fixation condition, which provided a stable baseline of awake, focused attention without external cognitive load.
- ***NF*** *Training (15 min)*: Participants completed three 5‐min training blocks with 1‐min breaks. The feedback threshold was adaptive to ensure continuous challenge and promote learning. The adaptive algorithm functioned as follows: at the end of every 30‐s epoch, the system calculated the participant's mean attention index. If this mean value exceeded the current threshold for three consecutive epochs, the threshold was automatically increased by 5% for the subsequent epoch. Conversely, if the mean index fell below the threshold for three consecutive epochs, the threshold was decreased by 5% to prevent frustration and maintain engagement. This dynamic adjustment ensured the task difficulty was continuously matched to the participant's current performance level.
- *Post‐Training Questionnaire*: Participants completed a brief survey describing any strategies they used (e.g., “I focused on my breathing,” “I rehearsed the text in my head”). This qualitative data was used to interpret individual differences in self‐regulation approaches but was not used to modify the protocol.

#### 2.4.2 Day 2‐4: Experimental Conditions

**FIGURE 2:**
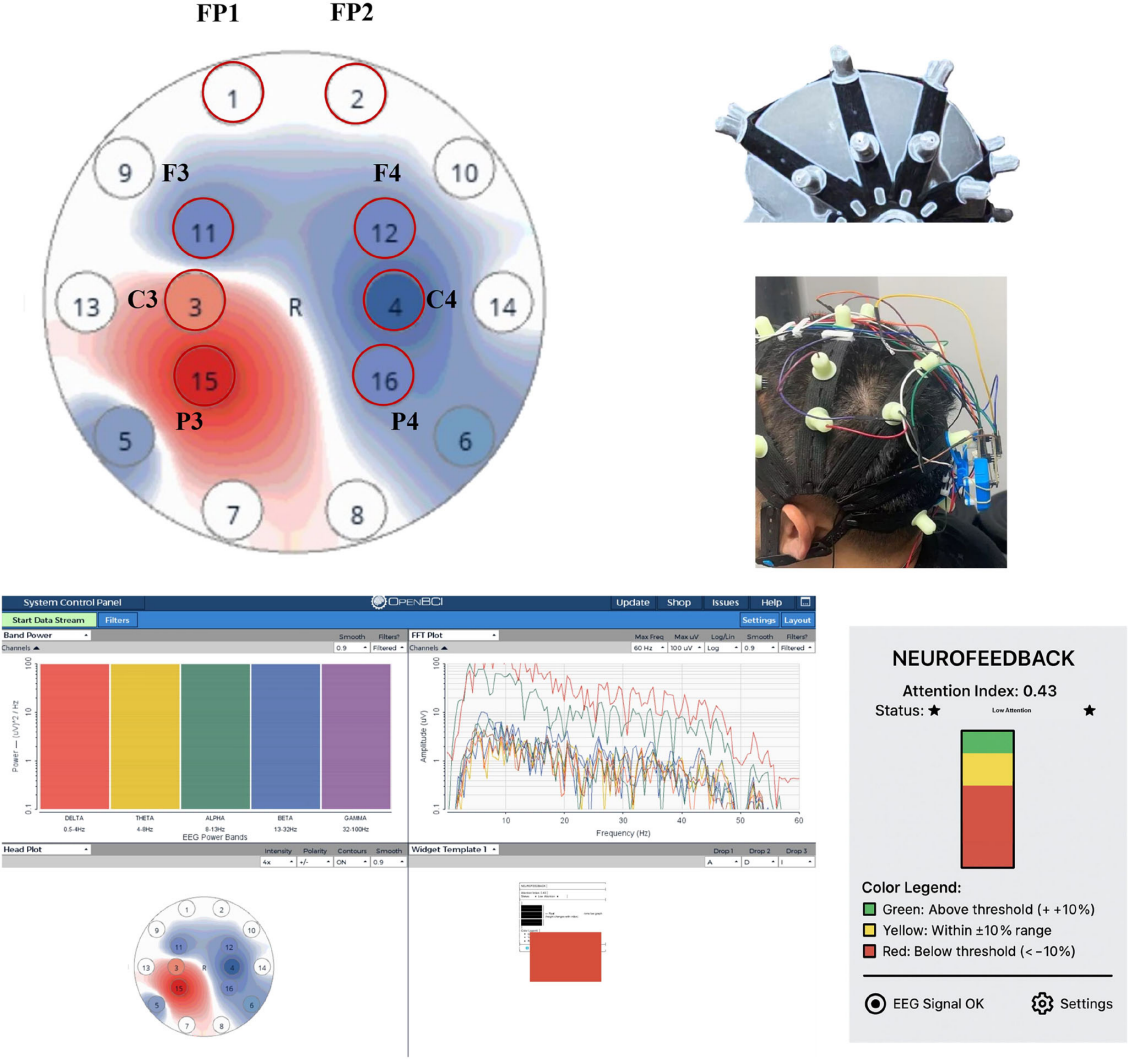
Real‐time neurofeedback interface based on attention index.

On Days 2–4, each participant completed the three experimental conditions (NF, Sham, Device Control) in a randomized, counterbalanced order. Each session adhered to an identical protocol:

1. *Pre‐task Baseline (2 min)*: EEG was recorded during an eyes‐open, fixation‐cross state to establish a pre‐learning baseline.
2. *Learning Phase (30 min)*: Participants engaged in a self‐paced Classical Chinese learning task. Instructional materials (texts, annotations, and explanations) were presented paragraph‐by‐paragraph on a computer screen.
  o NF Condition: The real‐time, dynamic attention index bar was displayed adjacent to the text.
  o Sham Feedback Condition: A static, high‐performance (green) bar was displayed, mimicking optimal feedback without actual EEG input.
  o Device Control Condition: The EEG equipment was worn, but no visual feedback was provided.
3. *Post‐learning Assessment*: Immediately following the learning phase, participants completed:
  o An *immediate comprehension test*.
  o The *Ancient Word Semantic Interference Task (AWSIT)*.
  o The *NASA‐TLX* subjective workload scale.
  o A *strategy‐use questionnaire* detailing the frequency and perceived effectiveness of attentional regulation strategies.
    EEG, behavioral performance (accuracy [ACC]), and response times (RT) were synchronously recorded throughout the entire session. Figure 2.

Illustration of the NF display used in the EEG training sessions. The interface features a dynamically updating vertical bar graph representing the participant's current attention index, calculated in real‐time using the Beta/(Alpha + Theta) ratio. The bar changes color based on performance: red indicates values below the individualized attention threshold, yellow reflects performance within ±10% of the threshold, and green signals values above the threshold.

### 2.5 Evaluation Metrics

To comprehensively assess the effects of EEG feedback on Classical Chinese learning and attentional regulation, multi‐level indicators were extracted across four dimensions: neural response, behavioral performance, subjective experience, and brain–behavior coupling.

At the neural level, the primary indicator was the EEG‐derived attention index, calculated in real‐time from Beta, Alpha, and Theta power features (channels: Fp1, Fp2, Cz), normalized to 0–100. In the data analysis phase, to exclude the interference of instantaneous outliers, all focus data during the learning phases were subjected to truncation within the ±2 standard deviation (SD) range and 5‐s sliding average smoothing. Subsequently, the mean focus level and stability (coefficient of variation, CV) were extracted for each condition.

At the behavioral level, two complementary cognitive tasks were administered: an Immediate Comprehension Test and an AWSIT. The comprehension test covered semantic recognition, contextual inference, and higher‐order semantic differentiation. Each subscore was computed separately and standardized to a 100‐point total. AWSIT assessed the ability to inhibit “modern meaning interference” using a 2 (judgment: yes/no) × 4 (condition) design with 160 trials. Trial types included true congruent, false incongruent, false unrelated, and false phonographic conditions. RT and ACC were recorded, and interference effects were quantified (e.g., FI_RT—FU_RT; FP_RT—FU_RT). By comparing participants’ reaction times and accuracy rates between the false incongruent condition (rejecting modern meanings) and the false unrelated condition (rejecting unrelated meanings), the cognitive cost of inhibiting dominant semantic interference was quantified to reveal the efficiency of conflict resolution in ancient word comprehension.

At the subjective level, participants completed the Chinese version of the NASA‐TLX after each session, providing VAS ratings (0–100) across six dimensions (mental demand, temporal demand, perceived performance, effort, frustration, and physical demand). A custom strategy‐use questionnaire also captured frequency and effectiveness ratings for attention‐regulation strategies, including open‐ended and Likert‐format items.

### 2.6 Statistical Analysis

Data were analyzed using repeated‐measures ANOVA and Pearson correlation analyses. Main outcome variables included the EEG attention index, comprehension scores, semantic interference task performance, and subjective workload ratings across the three conditions (NF, Sham, Device). Post hoc pairwise comparisons with Bonferroni correction were conducted following significant main effects. Correlation analyses were performed to explore brain–behavior relationships between EEG indices and behavioral outcomes. All statistical tests used a significance threshold of *p* < 0.05 and were conducted using SPSS 26.0.

## 3 Results

### 3.1 EEG‐Based Attention Index

**FIGURE 3:**
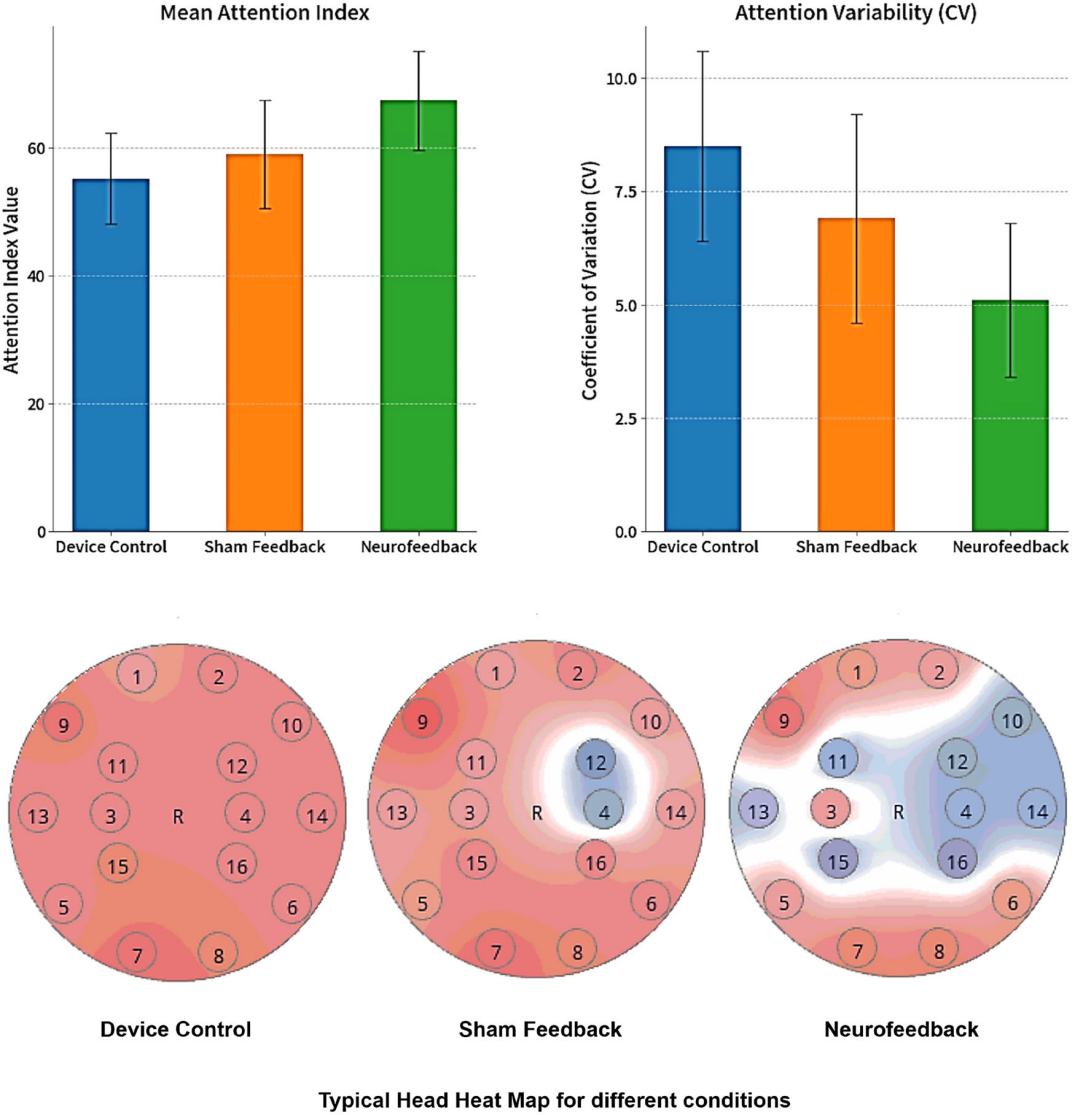
Mean attention index and attention variability across conditions.

A repeated‐measures ANOVA on the mean EEG‐derived attention index during the learning phase revealed a significant main effect of condition, F(2, 58) = 18.42, *p* < 0.001, *η* ^2^ = 0.388. Post hoc pairwise comparisons (Bonferroni‐corrected) showed that participants exhibited significantly higher attention levels in the NF condition compared to both the Sham (*p* < 0.001) and device control conditions (*p* < 0.001). The difference between the Sham and Device conditions was also significant (p = 0.042), indicating that pseudo‐feedback alone provided a modest attentional enhancement relative to passive device use. The attention curve in the NF condition was also more stable (lower CV), suggesting improved self‐regulation over time (see Table 1 and Figure 3).

**TABLE 1:** EEG‐based attention index during learning.

Measure	Device control	Sham feedback	Neurofeedback	*F*(2, 58)	*p*	*η* ^2^_*P*_
Attention index (mean)	55.2 ± 7.1	59.0 ± 8.4	67.4 ± 7.8	18.42	< 0.001	0.39
Attention variability (CV)	8.5 ± 2.1	6.9 ± 2.3	5.1 ± 1.7	9.06	< 0.001	0.24

### 3.2 Behavioral Performance

#### 3.2.1 Immediate Comprehension Test

Analysis of total comprehension scores revealed a significant effect of condition, F(2, 58) = 11.36, *p* < 0.001, *η* ^2^ = 0.281. Participants scored highest in the NF condition, followed by the Sham condition and the Device control condition. Pairwise comparisons indicated that the NF condition outperformed both Sham (p = 0.003) and Device (*p* < 0.001), while Sham also significantly outperformed Device (p = 0.041). Subscore analyses revealed the greatest NF‐related benefits in basic paraphrasing, applied analysis, and higher‐order semantics tasks (see Table 2).

**TABLE 2:** Comprehension test performance (total score = mean of three subscales).

Measure	Device control	Sham feedback	Neurofeedback	*F*(2, 58)	*p*	*η* ^2^_*P*_
Basic paraphrasing	74.1 ± 7.2	78.3 ± 76.7	84.6 ± 75.9	11.73	< 0.001	0.29
Applied analysis	67.8 ± 78.4	74.9 ± 77.5	82.3 ± 76.8	13.85	< 0.001	0.32
Higher‐order semantics	63.0 ± 710.1	78.5 ± 79.3	82.6 ± 77.1	17.42	< 0.001	0.37
Total score	68.3 ± 76.9	77.2 ± 76.2	83.1 ± 76.2	14.76	< 0.001	0.34

#### 3.2.2 Ancient Word Semantic Interference Task

For the semantic interference task (AWSIT), a series of one‐way ANOVAs revealed significant main effects of NF training condition across all critical measures. First, for reaction times on false incongruent trials, we observed a significant condition effect. Post hoc tests with Bonferroni correction indicated that the NF condition responded significantly faster than both the Device Control condition (*p* < 0.001) and Sham Feedback condition (*p* = 0.003). Similarly, False Phonographic trials showed a significant condition difference in RT, with NF participants demonstrating accelerated processing compared to Device Controls (*p* = 0.008). Most critically, the interference effect (modern‐meaning interference) exhibited the largest condition difference. NF training reduced interference effects by 46% relative to Device Control (*p* < 0.001) and by 40% compared to Sham Feedback (*p* < 0.001). Accuracy data paralleled these findings, with a significant condition effect on false incongruent trials. The NF condition achieved superior accuracy over both device control (*p* = 0.001) and sham feedback (*p* = 0.012) conditions (Table 3).

**TABLE 3:** Semantic interference task (AWSIT): reaction time and accuracy.

Measure	Device control	Sham feedback	Neurofeedback	*F* (2, 58)	*p*	*η* ^2^_*P*_
RT–false incongruent trials (ms)	782 ± 32	768 ± 29	714 ± 27	8.47	< 0.001	0.23
RT –false phonographic trials (ms)	745 ± 28	732 ± 26	703 ± 24	5.12	0.009	0.15
ΔRT false incongruent trials (ms)	52 ± 11	47 ± 9	28 ± 8	12.63	< 0.001	0.30
Accuracy –false incongruent trials (%)	78.2 ± 6.1	80.5 ± 5.7	86.3 ± 4.9	7.89	0.001	0.21

### 3.3 Subjective Measures

The NASA‐TLX total workload scores differed significantly across conditions, *F*(2, 58) = 8.92, *p* = 0.002, *η* ^2^_*P*_ = 0.24. Post‐hoc tests (Bonferroni‐corrected) indicated that NF produced significantly lower total workload than both sham feedback (*p* = 0.003) and device control (*p* < 0.001). Subscale analysis revealed significant reductions in mental demand (*p* < 0.001), effort (*p* = 0.004), and frustration (*p* = 0.008), while temporal demand, perceived performance, and physical demand showed no significant differences.

Strategy‐use analysis demonstrated significantly higher self‐rated effectiveness of attention‐regulation strategies during NF (*p* < 0.001), though strategy frequency did not differ across conditions (*p* = 0.31) (Table 4).

**TABLE 4:** Subjective cognitive load and strategy ratings.

Measure	Device control	Sham feedback	Neurofeedback	*F*(2, 58)	*p*	*η* ^2^_*P*_
NASA‐TLX total	65.7 ± 8.0	60.2 ± 7.4	48.3 ± 6.1	8.92	0.002	0.24
Mental demand	72.1 ± 9.3	66.4 ± 8.5	55.0 ± 7.2	9.41	0.001	0.25
Temporal demand	58.3 ± 10.2	56.7 ± 9.8	54.9 ± 8.3	0.85	0.432	0.03
Perceived performance	63.5 ± 7.5	60.8 ± 6.9	59.1 ± 6.3	1.24	0.297	0.04
Effort	68.5 ± 7.9	63.8 ± 7.0	52.4 ± 6.8	7.28	0.004	0.20
Frustration	61.2 ± 10.1	58.7 ± 9.3	46.5 ± 8.4	6.15	0.008	0.18
Physical demand	41.3 ± 6.5	39.8 ± 5.7	38.6 ± 5.2	0.92	0.404	0.03
Strategy effectiveness	4.1 ± 1.7	4.8 ± 1.5	6.6 ± 0.5	10.33	<0.001	0.26
Strategy frequency	5.0 ± 1.0	5.2 ± 1.1	5.5 ± 1.3	1.20	0.310	0.04

### 3.4 Brain–Behavior Correlation

**FIGURE 4:**
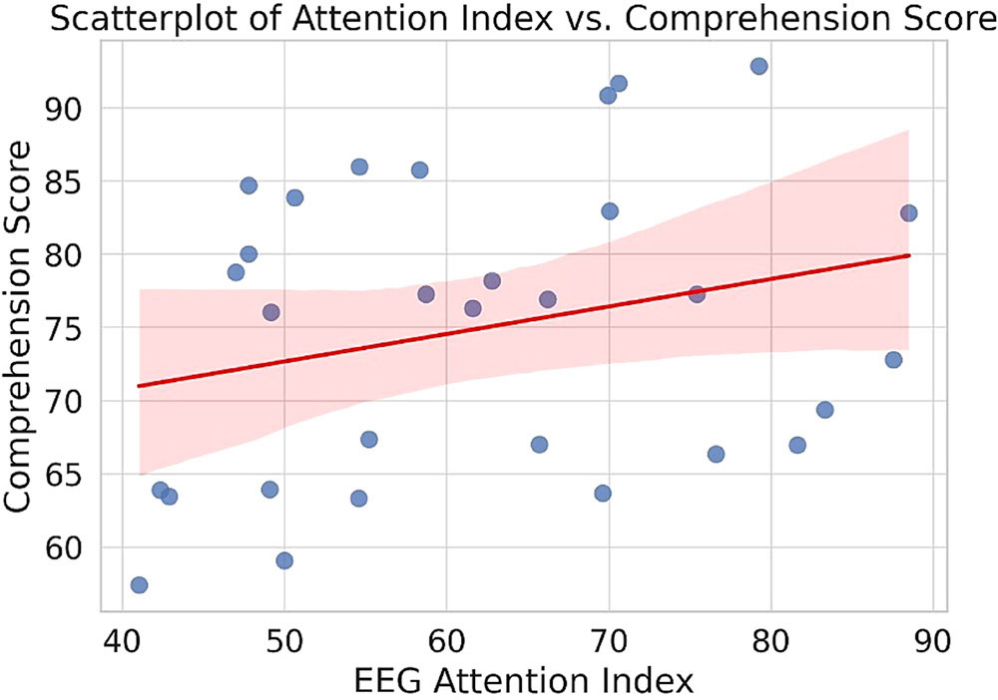
Scatterplot of attention index versus total comprehension score.

Pearson correlation analysis revealed a strong positive correlation between mean attention index and comprehension test total score in the NF condition (*r* = 0.63, *p* < 0.001), but not in the sham (*r* = 0.19, p = 0.108) or device control (*r* = 0.12, p = 0.237) conditions. No significant correlations were found for subjective workload and EEG metrics (Figure 4).

## 4 Discussion

This study reveals for the first time the acute modulatory effects of portable dry‐electrode EEG NF in classical Chinese learning through a three‐group repeated‐measures design. It was found that the NF group significantly enhanced the EEG‐based concentration index (β/(α+θ) ratio) and reduced the variability of attention (CV) compared to the pseudo‐feedback group (Sham) and the device control group (device control), a result that supports the specific role of NF in optimizing real‐time cognitive states (Nan et al. 2022). This finding is consistent with previous laboratory studies on the enhancement of working memory by NF, but the present study further extends its application scenario by confirming the acute facilitation of higher‐order language processing (e.g., semantic integration of ancient texts) by this technique (Pinti et al. 2020). Notably, the pseudo‐feedback group still demonstrated better attentional enhancement than the device control group, suggesting that the visual feedback interface may produce partial cognitive gains through expectancy effects (e.g., mental cues), which is in line with the previously proposed “neurofeedback placebo effect” hypothesis. However, the NF group had a significantly higher effect size (*η* ^2^ = 0.39) and lower attentional variability than the pseudo‐feedback group, suggesting that real neuromodulator mechanisms (e.g., coupling of β‐oscillatory enhancement and θ‐inhibition) play a dominant role in stabilizing cognitive resource allocation (Horowitz 2012). This finding provides important insights for educational neural engineering: in real classroom settings, the role of NF depends not only on the design of the feedback interface but also on its precise modulation of specific neural oscillation patterns.

The behavioral results of this study further confirm the acute promotional effect of NF on classical Chinese learning, as evidenced by the NF group achieving significantly higher total scores and subscale scores in the immediate comprehension test compared to the sham feedback group (sham) and the device control group (device control). This finding aligns with theoretical expectations in the field of cognitive neuroscience regarding the enhancement of language processing efficiency through NF (Marzbani et al. 2016), but it is the first to validate its differentiated beneficial effects on multi‐level semantic processing in a real‐world classical Chinese learning context (Duric et al. 2025). Notably, the NF group demonstrated the most significant advantage in higher‐order semantic tasks (*η* ^2^ = 0.37), suggesting that NF may enhance support for complex cognitive operations such as inhibiting conflicts between ancient and modern word meanings and contextual inference by optimizing functional coupling in the prefrontal‐temporal network (Pinto‐Orellana et al. 2024). From an educational application perspective, this hierarchical benefit effect (basic interpretation → application analysis → higher‐order semantics) holds significant pedagogical implications: it suggests that neural feedback not only accelerates the encoding of surface‐level language features (e.g., identifying function words) but may also enhance learners' ability to analyze deeper cultural connotations by improving the flexible allocation of cognitive control resources (Akhutina et al. 2024).

The subjective measurements and brain‐behavior association analysis in this study revealed the multidimensional benefits of NF in optimizing the classical Chinese learning experience. In terms of cognitive load, the NF group exhibited significantly lower NASA‐TLX total scores, particularly outperforming the sham feedback group (sham) and device control group (device control) in key dimensions such as psychological needs, effort level, and frustration. This finding aligns with theoretical hypotheses in educational neuroscience regarding the role of NF in alleviating cognitive load (L. C. Wang et al. 2025), but it is the first to confirm its regulatory effect on the emotional‐motivational dimension in the context of classical Chinese learning. Notably, although there were no group differences in strategy usage frequency, participants in the NF group reported significantly higher subjective evaluations of the effectiveness of attention regulation strategies, suggesting that NF may enhance metacognitive monitoring abilities (such as awareness of one's own cognitive state), enabling learners to utilize existing cognitive resources more efficiently (Mai 2024). More critically, brain‐behavior correlation analysis revealed a strong association between the attention index and comprehension scores under the NF condition (*r* = 0.63), whereas no significant correlation was observed in the sham feedback and device control groups. This result supports the specificity pathway hypothesis of “neurofeedback → attention optimization → behavioral gain” (Rahnev 2025). Future research should incorporate individual difference variables (such as baseline EEG characteristics or learning styles) to further elucidate the boundary conditions of this coupling relationship and provide theoretical foundations for personalized neuroeducation interventions.

Unlike conventional gel‐based systems, the dry‐electrode EEG setup employed in this study required minimal preparation time, offered user comfort during naturalistic academic tasks, and maintained acceptable signal fidelity for attention‐index extraction. Despite the traditionally noted limitations of dry EEG—such as higher impedance, motion artifacts, and restricted spatial resolution—our findings show that task‐relevant cognitive signals (e.g., frontal beta activity) can be reliably captured and used for real‐time feedback in cognitively demanding learning contexts. This supports emerging evidence on the validity of dry sensors for non‐clinical cognitive monitoring and adaptive feedback applications (Beauchemin et al. 2024; Kanna et al. 2024).

Crucially, the system successfully tracked and modulated participants' attentional states via a customized Beta/(Alpha+Theta) ratio, derived from frontal electrodes (Fp1,Fp2,F3,F4) known to reflect sustained attention and executive function. Only under the NF condition did participants exhibit significantly elevated attention indices and corresponding improvements in learning performance and cognitive control (M. S. Lee et al. 2025). These effects were absent in the sham and passive control conditions, indicating that the dry‐EEG‐based closed‐loop feedback system was critical in inducing neurocognitive change. The tight correlation between attention regulation and performance on the AWSIT further confirms that dry‐sensor EEG can capture behaviorally meaningful neural dynamics in real time (Sukumaran and Manoharan 2025).

From a systems and signal engineering perspective, this study supports the notion that wearable dry‐EEG BCIs can serve as effective cognitive support tools in real‐world educational settings. The low‐intrusion hardware design, Bluetooth‐enabled data streaming, and embedded signal processing pipeline demonstrate a feasible model of lightweight, deployable neurotechnology (Dritsas et al. n.d). Importantly, the system delivered responsive and personalized feedback without external amplification, gel preparation, or invasive procedures, making it scalable for routine use in classrooms or at home. The dry electrodes’ durability and low‐maintenance characteristics also suggest suitability for longitudinal deployments, such as in personalized learning programs or neuroadaptive tutoring systems (Heim et al. 2025; Lepold et al. 2024).

This work also contributes to the understanding of how sensor‐based cognitive augmentation can benefit high‐level humanistic learning, such as classical Chinese interpretation, which involves semantic disambiguation, historical‐linguistic context integration, and reflective analysis. By demonstrating that even short‐term NF delivered via dry EEG sensors can impact such tasks, we extend the application of sensor systems from typical attention‐training or gaming contexts into the domain of deep linguistic cognition and higher‐order learning (Sandua 2024; Drigas and Sideraki 2024)

The findings demonstrate the considerable practical potential of portable dry‐EEG NF for real‐world educational settings. The system's ease of use, minimal setup time, and lack of need for specialized expertise significantly enhance its feasibility for classroom implementation. The portability and rapid setup of the dry‐EEG system also address key scalability barriers for real‐world educational deployment. This addresses a major barrier of traditional NF, moving it from the laboratory closer to scalable practice. While the initial hardware cost is a consideration, the ability to conduct brief, group‐based sessions and the declining cost of consumer neurotechnology improve its cost‐effectiveness. Furthermore, observed variations in response highlight the role of individual differences in attentional control, suggesting that personalized NF approaches, tailored to a student's learning style and baseline cognition, could maximize future benefits. This aligns with the broader literature on educational NF, which seeks to develop practical tools for enhancing self‐regulated learning.

This study has several limitations that must be acknowledged. Firstly, the generalizability of the results is constrained by the participant sample of healthy university students, and the effects may not be directly applicable to younger populations, individuals with learning disabilities, or different cultural and educational contexts. Secondly, the temporal scope of the study is limited to acute effects; the long‐term efficacy and sustainability of a single NF session remain unknown and warrant investigation through extended training and follow‐up studies. Finally, while standard repeated‐measures analyses were employed, the study's statistical validation is limited by its sample size for detecting smaller effects and the exploratory nature of some comparisons, which should be confirmed in future pre‐registered research.

## 5 Conclusion

Our study demonstrates that dry‐electrode EEG NF significantly improves attention and semantic processing during Classical Chinese learning, addressing key limitations of prior work by (1) establishing ecologically valid protocols for dynamic classroom settings and (2) isolating NF‐specific effects from device‐related confounds. These findings advance the development of scalable, adaptive BCI systems for complex cognitive tasks.

Further research should optimize training protocols for long‐term retention and explore multimodal integration to enhance spatial resolution for deeper semantic processing analysis.

## Author Contributions

All authors contributed to the experimental design and writing the manuscript for this study.

## Conflicts of Interest

The authors declare no conflicts of interest.

## Ethics Statement

Written informed consent was obtained from all participants before the experiment, and the study protocol was approved by Shanghai University of Political Science and Law's ethics review board (Approval ID: 10277SPSL2025).

## Peer Review

The peer review history for this article is available at https://publons.com/publon/10.1002/brb3.70977.

## Data Availability

All data from this study are in the manuscript; please contact the corresponding author if you need anything else.
